# Removal of antibiotic resistance genes during livestock wastewater treatment processes: Review and prospects

**DOI:** 10.3389/fvets.2022.1054316

**Published:** 2022-12-22

**Authors:** Feng Huang, Yanting Hong, Chunhao Mo, Peier Huang, Xindi Liao, Yiwen Yang

**Affiliations:** ^1^College of Animal Science, South China Agricultural University, Guangzhou, China; ^2^Guangdong Provincial Key Lab of Agro-Animal Genomics and Molecular Breeding, and Key Laboratory of Chicken Genetics, Breeding and Reproduction, Ministry Agriculture, Guangzhou, China; ^3^National Engineering Research Center for Breeding Swine Industry, South China Agricultural University, Guangzhou, China

**Keywords:** antibiotic resistance gene, wastewater treatment process, livestock wastewater, remove, mechanism

## Abstract

Antibiotic resistance genes (ARGs) are emerging pollutants that have received extensive attention. Many different types of ARGs exist in livestock wastewater. If not effectively treated, they can threaten animal production, public health and the ecological safety of the surrounding environment. To address the high risk of livestock wastewater contamination by ARGs, the effects of different wastewater treatment processes on ARGs and their influencing factors and mechanisms are reviewed herein. Additionally, the current problems associated with removal of ARGs are discussed, and future research is proposed.

## Introduction

**Figure 1:**
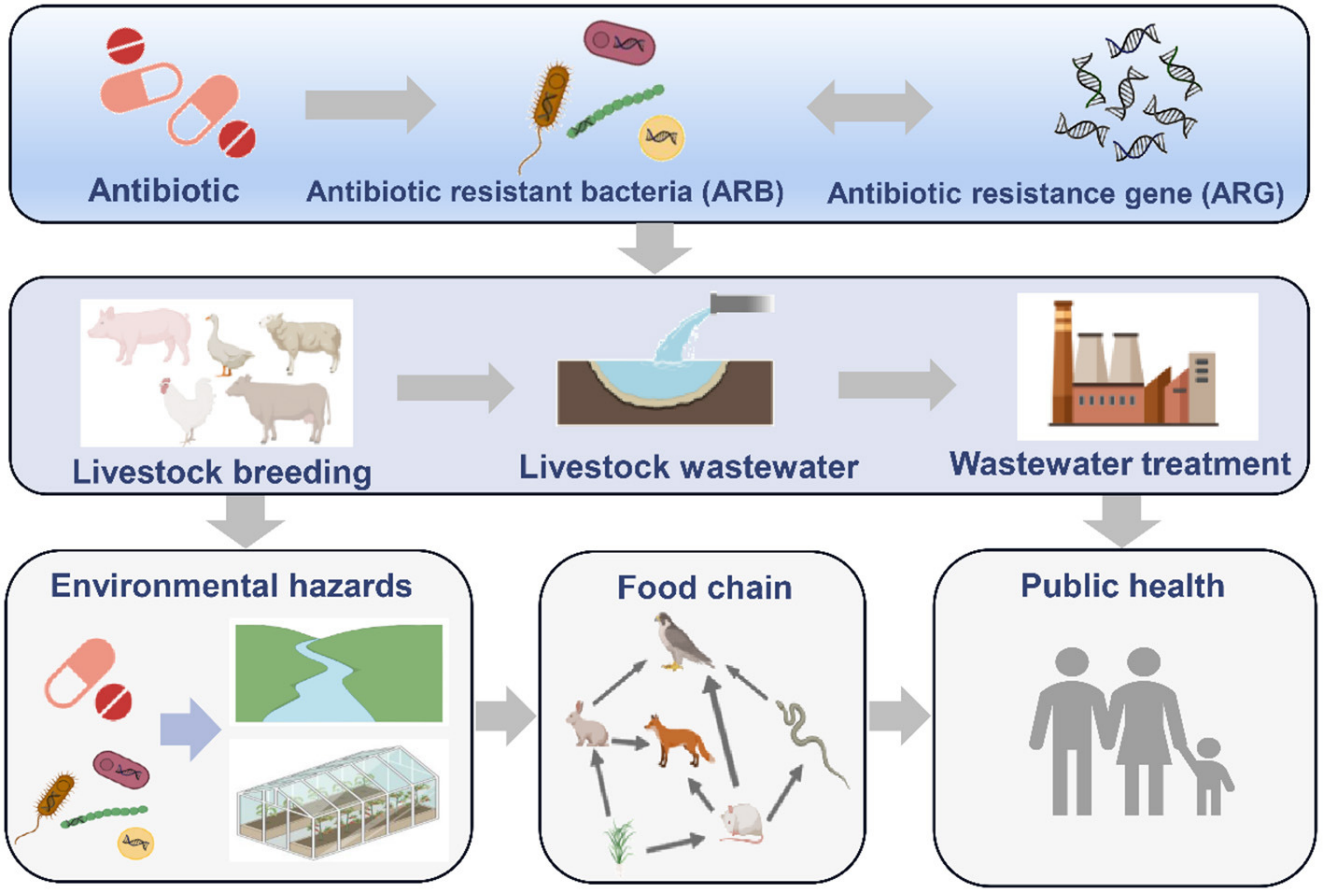
The fates of ARGs and ARBs in livestock and poultry farming environments.

Antibiotics are widely used in animal husbandry and bacterial drug resistance appears to be substantial (1, 2). Research results have shown that the total amount of antibiotics used in China in 2013 was ~162,000 tons, which accounted for half of global consumption, of which 52%, or 84,000 tons, were veterinary antibiotics (3). According to a report of the U.S. Food and Drug Administration (FDA), 29.9 million pounds of antibiotics were used with farmed animals in 2011, which accounted for 80.5% of the total antibiotic consumption in the United States (4). Similarly, in Vietnam, more than 11 million pounds of antibiotics were used for growth promotion, 25 million pounds for disease prevention and 37 million pounds for therapeutic purposes in the pig industry (5). Use of antibiotics may select among microorganisms harbored in animal intestines for resistant strains, carrying antibiotic resistance genes (ARGs) (6, 7). In addition, veterinary antibiotics cannot be completely absorbed or degraded by livestock and poultry, and most of the residual antibiotics are excreted through urine and fecal material (manure) in the form of unmetabolized drugs and their metabolites (8). These residual antibiotics promote enrichment of ARGs and their host bacteria in feces and wastewater (9). The highly abundant ARGs can enter the surrounding environment through manure or application of reclaimed water. Moreover, the high abundant ARGs also enter other sensitive bacteria through horizontal gene transfer and threaten ecological security (10, 11) (Figure 1). In addition, pathogens are also important hosts of ARGs, and they cause widespread and severe problems duo to antibiotic-resistance contamination (12). In 2019, about ~ 4.95 million deaths worldwide were related to infections with antibiotic-resistant bacterial, of which 1.27 million people died directly from antibiotic resistance (13). The use of antibiotics as growth promoters have been banned in an increasing number of countries. However, the copy numbers of ARGs remain elevated in animal raising environment. Therefore, it is necessary to implement effective removal processes to prevent and control further spread of antibiotic resistance. Wastewater is one of the main livestock wastes and an important repository for ARGs (14). China produces ~ 3.8 billion tons of livestock waste every year (15). Among this, aquaculture wastewater exceeded 460 million tons (16). However, current livestock wastewater treatment processes are mainly aimed at treatment of conventional pollutants such as organic matter, ammonia nitrogen, phosphorus, antibiotics and heavy metals, but not ARGs or antibiotic resistant bacteria. Therefore, it is necessary to understand the ARGs removal effectiveness of different livestock wastewater treatment processes, clarify their influencing factors and mechanisms, and provide reference for prevention and control of ARGs and antibiotic-resistance pollution.

## Profile of ARGs in livestock wastewater

### Abundance of antibiotic resistance genes in livestock wastewater

Livestock wastewater is an important repository for ARGs. Tetracyclines, aminoglycoside, sulfonamide, macrolide, streptomycin, bacitracin, β-lactam, chloramphenicol, quinolone, trimethoprim, fosmidomycin, polymyxin, and vancomycin resistance genes are frequently detected in wastewater, and the absolute abundance of ARGs is between 10^8^ and 10^10^ copies/mL, with a relative abundance between 10^−3^ and 10^−1^ copies/16S rRNA genes (10, 17–19). Among them, the abundance of tetracycline, aminoglycoside, macrolide–lincosamide–streptogramin (MLS), sulfonamide and chloramphenicol resistance genes were the highest, and they account for 28.13, 23.64, 12.17, 11.53, 8.74 and 6.18% of total ARGs respectively, and their abundances can be up to 2.41 × 10^−1^, 2.03 × 10^−1^, 1.04 × 10^−1^, 9.90 × 10^−2^, 7.49 × 10^−2^ and 5.31 × 10^−2^ copies/16S rRNA genes, respectively (19). Based on different resistance mechanisms, ARGs can be divided into efflux pump, target protection (ribosome protection), enzyme modification (antibiotic inactivation) and other types of resistance genes (20–22). In addition, most ARGs exist within bacteria in livestock wastewater, while some exist in phages, and small amounts of ARGs are freely present in wastewater in the form of nucleic acids. One study reported that the absolute abundances of ARGs in bacteria and phages were 10^9^ and 10^6^ copies/mL, respectively (23).

### Hazards associated with ARGs in livestock wastewater

Residual ARGs in livestock wastewater can enter farms and surrounding environments with efflux and utilization of the wastewater and threaten human health (9, 24). Yang et al. (10) found that the abundance of the *bla*_*TEM*_ resistance gene in the downstream river water around a pig farm was 10^7^ copies/mL, which was significantly higher than that in the upstream river water (10^5^ copies/mL). In addition, the abundance of ARGs in downstream sediments around pig feedlots was 1.2-fold higher than that in the upstream sediments (25). This means that the ARGs that remained in livestock wastewater were enriched in the surrounding water and soil, and they can be carried by bacteria or phages, enter the bodies of aquatic animals and plants, and cause more severe antibiotic-resistance contamination throughout the food chain (26). Moreover, ARGs and resistant bacteria present in livestock wastewater can also enter the air in the form of aerosols (27). Sun et al. (28) found that the composition of ARGs in the fecal samples of students during 3-month internships at swine farms were consistent with that in the pig farm environment, indicating that the ARGs can enter the human gut through certain means and affect the composition of ARGs in the human gut. It should be noted that high-risk ARGs such as *mcr-1, bla*_*NDM*_ and *tetX* were detected in livestock wastewater (29). ARGs such as *bla*_*CTX*_, *bla*_*CMY*_and *qnrB*, which have clinical resistance risks, were also detected in swine wastewater, and these can confer antibiotic resistance in pathogenic bacteria, making the treatment of infectious diseases more difficult (30, 31). Therefore, it is urgent to pay attention to the high abundance of residual ARGs in livestock waste.

## Efficacy of ARG removal by livestock wastewater treatment processes

### Biological treatment processes for removal of ARGs

#### Anaerobic treatment

Anaerobic treatment is a treatment technology that decomposes organic matter to generate biogas under anaerobic conditions with the help of anaerobic microorganisms. The four stages, hydrolysis, acidification, hydrogen and acetic acid production, and methane production, are completed by different anaerobic microorganisms (32). The up-flow anaerobic sludge blanket (UASB), anaerobic baffled reactor (ABR), buried biogas digester (BBD) and anaerobic filter (AF) are commonly used for treatment of livestock farm wastewater (33, 34). Another study showed that anaerobic treatment effectively reduced ARGs, among which *tetX, ermB, mefA, ermF* and *sul2* showed average reductions of 1.34, 1.07, 1.03, 0.83, and 0.72 log, respectively (35). The removal rates of BBD and UASB anaerobic treatments based on the relative abundance of ARGs were 3.53 and 71.02% in another study, respectively (34) (Table 1). However, other studies have also shown that anaerobic treatments cannot effectively remove ARGs, and the relative abundances of bacterial ARGs were not decreased significantly after treatment (23, 36). Aerobic treatment refers to denitrification by facultative anaerobic denitrifying bacteria in an anaerobic or aerobic state (dissolved oxygen DO < 0.5 mg/L). Aerobic treatment is similar to anaerobic treatment, and has a certain removal effect on ARGs. However, some functional microorganisms, such as anaerobic denitrifying bacteria, are ARG hosts (37). They may increase the abundance of ARGs in anaerobic tank, which may increase the risk of environmental pollution.

**Table 1:** Effects of common livestock and poultry wastewater treatment processes on antibiotic resistance genes removal.

**Process types**		**Removal**	**Remark**	**References**
Biological treatment	Anaerobic	The removal efficiencies for the relative abundance of total ARGs were 3.53 ~ 71.02%, while *tetG, mdtB* and *mdtC* enriched.	Denitrifying bacteria such as denitrifying bacteria are host bacteria for ARGs	(34)
	Aerobiotic	The total ARG abundances decreased by log 1.77. The relative abundance of most genes (except *sul2* and *mecA*) decrease.	–	(40)
	Short-path nitrification and denitrification	The average abundances of ARGs reduced from 10^9^ copies/mL to 10^8^ copies/mL.	–	(23)
	Artificial wetland	The absolute total ARG concentrations were reduced by 0.7–1.24 log.	The removal effect is affected by the type of constructed wetland and plant species, etc.	(41)
	Microalgae system	The relative abundance of *qnrA, tetW, qnrS* and *intI1* genes were decreased ranging 0.62 to 3 log. While the *sul1* gene was increase.	The removal rate of resistant bacteria is 88.5%	(43)
Physical treatment	Coagulation	The maximum removal of ARGs was 3.1 log.	Including polyaluminum chloride and polyferric sulfate, etc.	(46)
	Membrane separation	A reduction of *vanA* and *blaTEM* by 0.9, 3.6 and 4.2 log for membranes with100, 10 and 1 kDa, respectively.	Ultrafiltration membranes, etc.	(52)
Chemical treatment	Fenton	The maximum log reductions of *tetX* and *tetG* were 3.79 and 2.58 logs, respectively.	Including ${\text{Fe}}^{2+}/H_{2}O_{2}$ and UV/ H_2_*O*_2_ process.	(53)
	Chlorination, ultraviolet, and ozonation disinfection	1.68–2.55 log reductions of ARGs.	Including chlorination, ultraviolet (UV), and ozonation disinfection processes.	(54)

#### Aerobiotic treatment

Certain methods and equipment are used to force air into the wastewater so it is oxygenated by contact with the air, and the liquid is stirred to accelerate oxygen transfer from the air to the liquid. Aeration prevents the wastewater suspension from sinking and strengthens the contacts among organic matter, microorganisms and the dissolved oxygen in the wastewater. When sufficient dissolved oxygen is present, aerobic microorganisms oxidatively decompose the organic matter in wastewater. Studies have shown that aeration was beneficial for the reduction of ARGs (38, 39). Another study found that the abundance of total ARGs was decreased by 1.77 log copies/mL during aeration of the tank, but a change in *sul1* abundance was not observed (40).

#### Other biological treatments

In addition to the above processes, there are also biological treatment processes such as constructed wetlands, short-cut nitrification and denitrification, and microalgae-bacteria symbiosis technology. Constructed wetland is primarily divided into surface flow constructed wetland (SFCW), horizontal subsurface flow wetland (HSFW) and vertical subsurface flow wetland (VSFW). It was found that the efficiencies for removal of *tetM* from wastewater by constructed wetlands was 70.9–97%, the removal rates for *sul1* was 49.5–92.9%, and the removal rate for *ermB* was 58.2–96.7% (41). The short-path nitrification and denitrification process showed substantial levels of nitrogen and phosphorus removal. The average abundance of bacterial/phage ARGs in the effluent of a short-range nitrification/denitrification system was significantly lower than that in the influent (23). After treatment with microalgae-bacteria, the abundance of *bla*_*TEM*_ and *ermB* in wastewater decreased by 0.56 and 1.75 log, respectively (42). In a microalgae treatment system, the relative abundance of *qnrA, tetW*, and *qnrS* decreased significantly, but the relative abundance of *sul1* increased (43).

### Physical treatments for removal of ARGs

In a pig farm wastewater treatment system, the physical treatment processes mainly include coagulation and membrane separation technology. Under the action of a coagulation, the suspended solids present in wastewater are aggregated into larger particles and precipitate, thereby removing many suspended solids from the wastewater and achieving wastewater treatment. Chemical coagulants include polyaluminum chloride, polyaluminum sulfate, aluminum hydroxide iron, polyferric chloride and ferric chloride (44, 45). Studies have shown that treatment by coagulation and sedimentation provide successful removal of most ARGs in wastewater and that the abundance of ARGs can be reduced by 0.5–4.7 log (46, 47). However, different coagulants also have different effects on ARGs removal. The average removal of *sul1* by polyferric chloride was 2.3 log, and the average removal of *sul1* by ferric chloride was 2.6 log.

Membrane separation is a method used to separate, purify and concentrate different components through selective separation. In wastewater treatment, membrane separation is mainly achieved by using pressure as a driving force with the pore size, electrostatic effect, and diffusion effect of the membrane (48). These processes mainly include reverse osmosis (RO), nanofiltration (NF), microfiltration (MF), and ultrafiltration (UF), which have better removal capacities with ARGs (49–51). The removal rate of ARGs by membrane separation is inversely proportional to the membrane pore size (52).

### Chemical and physicochemical treatments for the removal of ARGs

Chemical treatment processes include ozonation, chlorination and Fenton oxidation. Physicochemical mainly include UV irradiation and electrolysis. Many studies have shown that chemical oxidations remove ARGs from livestock wastewater. It was found that Fenton oxidation reduced selected ARG levels by 2.58 ~ 3.79 log (53). With an ozone mass concentration of 177.6 mg/L, ARGs decreased by 1.68 ~ 2.55 log; under a UV dose of 12.477 J/cm^2^, ARGs decreased by 2.48 ~ 2.74 log (54). With UV doses of 249.5 and 62.4 mJ/cm^2^, *tetX* decreased by 0.58 and 0.23 log, respectively (3). Studies have shown that microbial fuel cells constitute promising alternatives for enhanced removal of ARGs (55, 56). Recently, a study showed that the relative abundance of *sul1* in the biofilm and effluent of microbial fuel cells ranged from 4.70 × 10^2^ to 7.80 × 10^1^, and the relative abundance of *sul2* ranged from 4.21 × 10^5^ to 7.61 × 10^3^, which was lower than those in natural water bodies and in drinking water (57).

## Factors influencing ARGs removal during livestock wastewater treatment processes

The factors influencing ARG removal are divided into direct driving factors and indirect influencing factors. The direct driving factors include factors that directly affect changes in ARGs, such as microbial communities, mobile genetic elements (MGEs), and wastewater physicochemical factors (58). Indirect influencing factors refer to those that indirectly remove of ARGs through factors affecting wastewater quality and microbial communities, such as breed management and operating parameters.

### The direct driving factors that affect the changes in ARGs in livestock wastewater

#### Microbial communities

Microbial communities, including those of bacteria, viruses (bacteriophages), archaea and fungi, are the main direct drivers of ARG changes. Among them, bacteria are also important ARG hosts. Most of the ARGs present in livestock wastewater exist in bacterial cells. Studies have shown that *Escherichia coli* in livestock wastewater is a host for a variety of ARGs, including tetracycline, aminoglycoside, MLS, β-lactam and sulfonamide resistance genes (59–61). *Enterococcus* is the main host for vancomycin resistance genes, and *Klebsiella* and *Enterobacter* are hosts for fosfomycin resistance genes (62). In addition, another study showed that phages also carry a high abundance of ARGs, including multiple types of ARGs, reaching 2.01 ± 0.21 log (23). The microbial community acts as a carrier for ARGs. Therefore, the ARG composition changes with changes in the composition of the microbial community (11, 58). ARGs are spread by the movement of microorganisms. Therefore, during treatment of livestock wastewater, changes in the composition of the microbial community directly affect the process of ARG removal. The more complex the host composition of ARGs in livestock wastewater, the more difficult they may be to remove.

#### Mobile genetic elements

MGEs mainly include insertion sequences, transposons, integrons, plasmids, and bacteriophages (63, 64). The ARGs in livestock wastewater can be horizontally transferred by MGEs (65, 66), such that ARGs can be transferred among the same or even different microorganisms (67). Due to horizontal transfer of ARGs, microorganisms that do not originally have antibiotic resistance can acquire antibiotic resistance, which increases the complexity of antibiotic-resistant contamination in livestock wastewater (68, 69). Transposons can be copied or broken off from the original site, then cyclized and inserted into other sites. Integrators can capture and integrate foreign genes with their unique structure. Plasmids can carry genes and spread them among different cells.

#### Physicochemical factors

Here, physicochemical factors mainly refer to the physicochemical properties of livestock and poultry breeding wastewater and include temperature, pH, electrical conductivity (EC), total nitrogen (TN), total phosphorus (TP), ammonia nitrogen, nitrate nitrogen, organic matter and heavy metals, which indirectly affect antibiotic resistance mainly by affecting the compositions of microbial communities. Studies have shown that total nitrogen, ammonia nitrogen and total phosphorus were significantly and positively correlated with the abundance of *sul1, sul2, tetA, tetB, tetC* and *qnsR* (23, 70, 71); total organic carbon was significantly and positively correlated with *fexA, fexB, cfr, sul1, tetW, tetO, tetQ* and *tetS* (72). In addition, physicochemical factors can indirectly affect the abundance of ARGs by affecting the morphology of free ARGs. One study showed that temperature affected the degradation efficiency of free nucleic acids, and free nucleic acids decayed as the temperature was increasesed (73).

### Indirect influencing factors that affect ARGs removal during treatment of livestock and poultry wastewater

#### Breed management

Breed environment, manure removal methods, disinfection methods and other operations in breed management affect the removal of ARGs from livestock and poultry breeding wastewater. Antibiotics and heavy metals are widely used in animal husbandry (74–76). However, antibiotics and heavy metals that cannot be fully absorbed and metabolized can lead to antibiotic-resistance contamination (77, 78). A recent study found that when the ambient temperature decreased, the abundance of ARGs in feces and cecum contents decreased significantly (79). Common methods of manure removal in farms include artificial dry manure removal and mechanical manure removal. Among them, artificial dry manure removal has the best effect on the removal of ARGs (23). During disinfection processes in farms, disinfectants have a certain reduction effect on free ARGs and resistant bacteria. For example, chlorine disinfection can effectively kill resistant bacteria, but it cannot effectively remove ARGs (80). In addition, a recent study showed that after the use of disinfectants, the chloramines and free chlorine present in the wastewater increase the conversion rates of free ARGs (81).

#### Treatment parameters

Different treatment parameters for wastewater treatment process, such as water intake, residence time, temperature and aeration amount, affect the treatment effect of livestock and poultry breeding wastewater, and thereby affect the quality of the effluent (physicochemical factors such as total nitrogen, ammonia nitrogen, total phosphorus and organic matter). These physicochemical factors can indirectly affect the removal of ARGs by affecting the composition of the host flora (77, 82), and they can also directly affect the removal of free ARGs. In addition, treatment parameters such as temperature and the aeration rate can also directly or indirectly affect the removal of ARGs. Diehl (83) found that the abundance of *tetA, tetO, tetW*, and *tetX* decreased with increasing temperature in anaerobic reactors. However, it was found that although the ARG abundances in wastewater treatment systems were higher in winter than in summer, the removal rates ARGs such as *tetG, tetM*, and *tetX* were higher in winter than in summer (84).

## Mechanisms for removal of ARGs in livestock wastewater treatment processes

**Figure 2:**
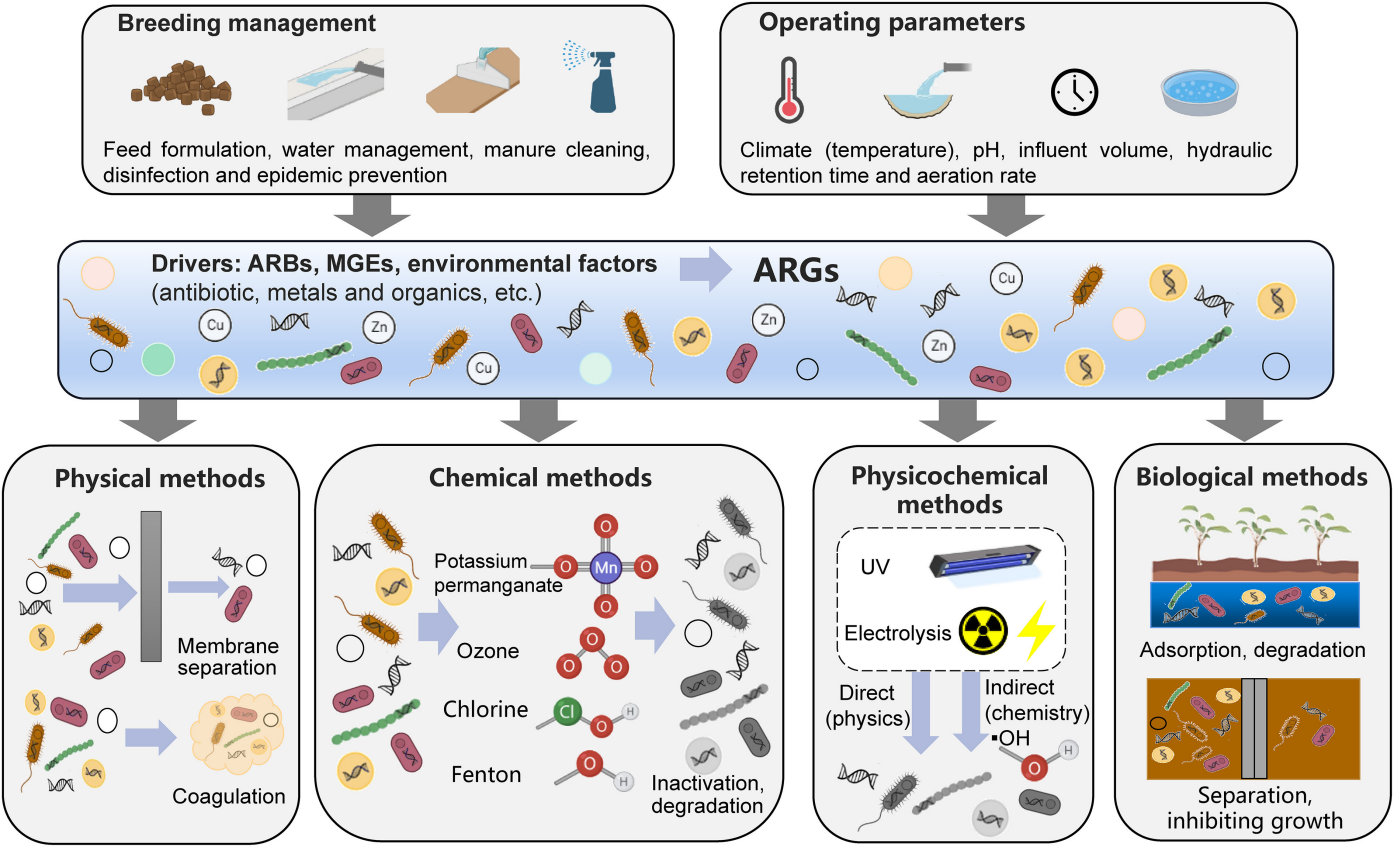
Removal mechanisms for antibiotic resistance genes from livestock wastewater treatment processes.

Based on previous research, this review summarizes the removal mechanisms active in common wastewater treatment processes in livestock farms in China; these include physical, chemical, physical-chemical and biological removal mechanisms (Figure 2) (85).

### Removal mechanisms for physical methods

The ARGs in livestock wastewater are mainly derived from livestock manure. Physical methods mainly refer to the technical processes of coagulation sedimentation and membrane separation to separate the larger solid residues from livestock wastewater, thereby reducing the abundance of ARGs. Therefore, when a physical method is used to separate solid residues such as feces, most of the ARGs that remain in the feces will be separated to achieve ARG removal, but this method cannot remove free ARGs and their hosts in the wastewater. In addition, filters of small pore sizes (< 0.22 μm) can retain bacteria carrying antibiotic resistance gene in wastewater. The removal rate of ARGs is inversely proportional to the membrane pore size. In addition, the removal effects of different filter membrane materials on ARGs are also different (85, 86). The removal effect of polyethersulfone ultrafiltration membranes on ARGs is better than that of polyvinylidene fluoride ultrafiltration membranes.

### Removal mechanisms for chemical and physicochemical and methods

Chemical oxidation is a method that reduces ARGs by decomposing antibiotic-resistant bacteria through strong oxidants. There are two mechanisms of chemical oxidation. In one, the oxidants react with antibiotic-resistant bacteria, dissolve cell walls and cell membranes, and then react with the cytoplasm and nucleic acid substances to kill bacteria. SecondS, the oxidants produce hydroxyl radicals, which increase pH and decompose resistant bacteria (87, 88). Ozonolysis produces large number of hydroxyl radicals, which react with components such as peptidoglycan in the cell membrane and cell wall, destroy the cell envelope structure, change cell permeability, and then degrade intracellular DNA and remove ARGs (87, 89). Chlorine alters the permeability of cells by disrupting cell surface structures, allowing their internal components to be broken down. At the same time, chlorine decompose into ClO^−^ and HClO, which can enter antibiotic-resistant bacteria and further oxidize components such as nucleic acids (90, 91).

Physical-chemical methods mainly include ultraviolet methods and electrolysis methods. Ultraviolet light can be absorbed by intracellular or extracellular photosensitizers of antibiotic-resistant bacteria, producing large amounts of hydroxyl radicals and then oxidizing various cellular components to kill bacteria (54). In general, the UV intensity is proportional to the removal rate of ARGs (3). In addition, the use of UV in combination with other oxidants, such as Fenton and H_2_O_2_ can further improve the removal rate of ARGs (92, 93), which may be because the combination of different oxidation reactions can generate higher concentrations of oxidative radicals to improve oxidation efficiency. Iron-carbon microelectrolysis is commonly used, which uses the potential difference generated by iron and carbon components to form multiple tiny galvanic cells in water to achieve redox reactions and the coagulation, adsorption, and electrodeposition of pollutants and antibiotic-resistant bacteria (94, 95).

### Removal mechanisms for biological methods

Biological methods include processes such as anoxic/aerobic processes, short-range nitrification and denitrification processes, constructed wetlands and microalgae-bacteria symbiosis. Biofilms or activated sludge in anoxic/aerobic processes and short-range nitrification and denitrification processes can adsorb suspended solid organic matter and other substances in livestock wastewater and simultaneously remove nitrogen, phosphorus and other substances necessary for microbial growth. Therefore, they can not only isolate ARG host bacteria that are attached to solid fecal residues in the wastewater by membrane separation and sedimentation but also inhibit the growth of ARG hosts by reducing nutrients to realize the removal of ARGs. The removal of pollutants in wastewater from constructed wetlands is achieved through the synergistic action of plant roots and rhizomes, microorganisms and solid medium components. These different fractions provide different microenvironments for various removal processes, such as mechanical filtration, adsorption, photolysis, volatilization, chemical degradation, plant uptake and microbial metabolism (96). Among them, adsorption, chemical degradation, and plant uptake play important roles in the removal of ARGs, but the mechanism for plant uptake of ARGs is still unclear and needs further research (97). The removal effect of constructed wetlands on ARGs is affected by the type of constructed wetland and plant species. Microalgae mainly utilize nitrogen and phosphorus in wastewater through photosynthesis to reduce the total bacterial abundance of the wastewater, thereby reducing the abundance of individual ARGs (43). However, it should be noted that microalgae may adsorb individual bacteria, which may lead to the enrichment of individual ARGs (42, 43).

## Conclusion and prospects

Different processes have different removal effects on ARGs. Removal mainly proceeds via physical, chemical and biological mechanisms, which are influenced by major drivers such as microbial communities, MGEs and physicochemical factors. However, current treatment processes are mainly aimed at removal of conventional pollutants such as COD, ammonia nitrogen and total phosphorus and are not designed for removal of ARGs. Therefore, the current wastewater treatment processes cannot completely remove ARGs from wastewater, and some individual processes even have the potential to increase the abundance of individual ARGs. More importantly, overuse of antibiotics can produce arb and arg. Therefore, proper control of antibiotic usage in animal husbandry is an important way to reduce the spread of ARBs and ARGs through wastewater and feces. Future research on the removal of ARGs from livestock wastewater is anticipated to show the following trends:

(1) The focus of current research is to demonstration of ARGs and specific mechanisms for different treatment processes. At present, the rates and main mechanisms for removal of ARGs by typical wastewater treatment processes have been elucidated, but the removal effect and specific removal mechanisms of individual processes are still unclear.
(2) While paying attention to the removal of ARGs, it is necessary to pay attention to the impact of the process on the removal of total nitrogen and other substances and on the reduction of greenhouse gas emissions, and their relationship to ARGs. Total nitrogen removal and carbon emissions reduction will become new requirements for livestock wastewater treatment. However, the influence and relationships between total nitrogen removal, greenhouse gas emissions reduction and the removal of ARGs is yet to be clarified.
(3) Improving ARGs removal by existing wastewater treatment processes will be the focus of future research, and development of new processes will be an important trend. Currently employed wastewater treatment processes will be difficult to replace with new processes on a large scale and within a short period of time. Upgrade and transformations of the original processes are the most cost-effective options.
(4) The risk assessment system for antibiotic resistance must be improved to assess ARG removal more accurately. At present, the risk for contamination by antibiotic resistance genes is mainly assessed by analyzing the abundance of ARGs. The resistance characteristics of ARGs are also affected by genetic factors and environmental factors, and as-yet unknown influencing factors may exist that could affect the accuracie of ARGs contamination assessment.

## Author contributions

FH, YH, XL, and YY conceptualized the review. All authors contributed to the article and approved the submitted version.
